# Comparison of data from the juvenile dermatomyositis national (UK & Ireland) cohort biomarker study and repository for idiopathic inflammatory myopathies with a survey of current practice throughout the UK and Ireland

**DOI:** 10.1186/1546-0096-9-S1-P57

**Published:** 2011-09-14

**Authors:** N Martin, LR Wedderburn, JE Davidson, L Beard, CA Pilkington

**Affiliations:** 1 Great Ormond Street Hospital, London, UK; 2 Royal Hospital for Sick Children(Yorkhill) Glasgow, UK

## Background

There is no published evidence on how JDM is currently treated in UK and Irish paediatric rheumatology centres.

## Aim

To confirm whether current treatment agrees with physician’s perception of their treatment practices.

## Methods

We compared the results of a survey of current practice throughout the UK and Ireland with data prospectively collected since January 2000 from the UK JDM cohort study. Patients were classified as having mild, moderate or severe disease at presentation according to their Physicians Global Assessment (PGA) at diagnosis.

## Results

Fifteen of 16 Paediatric Rheumatology centres responded to the survey. For initial treatment of patients with mild disease 13/15 centres report using a combination of steroids and methotrexate and 8/15 would also use IV methylprednisolone. For moderately severe disease all centres use steroids and methotrexate and 11/15 also use IV methylprednisolone. With severe weakness, ulceration or systemic disease all centres use IV methylprednisolone as initial treatment, 13/15 use methotrexate and 9/15 use cyclophosphamide.

Prospective data were analysed from 98 patients in the UK JDM cohort study. A combination of steroids and methotrexate was used in the first year of treatment in 78% of patients with mild disease (PGA 0-3.3cm), 89% of patients with moderately severe disease (PGA 3.4-6.7cm) and 100% of patients with severe disease (PGA 6.8-10cm). Cyclophosphamide was used within the first year of diagnosis in 16% of patients with mild disease at presentation, 19% of patients with moderately severe disease and 38% of those with severe disease (p=0.05). There was no significant association between severity of disease at diagnosis and the use of intravenous steroids.

## Conclusions

Our study confirms that steroids and methotrexate form the basis of first line therapy in JDM throughout the UK and Ireland. Cyclophosphamide is commonly used for patients with severe disease, systemic involvement or cutaneous ulceration, suggesting that a consensus could be reached on treatment protocols within the UK.

